# Neuroendocrine Carcinoma of Uterine Cervix: Stereotactic Radiotherapy for Brain Metastasis and Stereotactic Body Radiotherapy for Renal and Pancreatic Metastases

**DOI:** 10.7759/cureus.8869

**Published:** 2020-06-27

**Authors:** Yoshimasa Mori, Yoshihisa Kida, Yasuhiro Matsushita, Shinichiro Mizumatsu, Manabu Hatano, Noriko Morita, Toyonori Tsuzuki

**Affiliations:** 1 Radiation Oncology and Neurological Surgery, Shin-Yurigaoka General Hospital, Kawasaki, JPN; 2 Radiology and Radiation Oncology, Aichi Medical University, Nagakute, JPN; 3 Neurological Surgery, Ookuma Hospital, Nagoya, JPN; 4 Neurological Surgery, Aoyama General Hospital, Toyokawa, JPN; 5 Gamma Knife Center, Ookuma Hospital, Nagoya, JPN; 6 Neurological Surgery, Gamma Knife Center, Ookuma Hospital, Nagoya, JPN; 7 Neurological Surgery, CyberKnife Center, Aoyama General Hospital, Toyokawa, JPN; 8 Neurological Surgery, Radiosurgery, CyberKnife Center, Aoyama General Hospital, Toyokawa, JPN; 9 Gynecology, Aichi Medical University, Nagakute, JPN; 10 Surgical Pathology, Aichi Medical University, Nagakute, JPN

**Keywords:** neuroendocrine carcinoma, uterine cervix, stereotactic body radiotherapy, chemotherapy, pancreatic, renal, brain, metastasis, stereotactic radiosurgery, stereotactic radiotherapy

## Abstract

A case of cervical neuroendocrine carcinoma (NEC) of the uterine cervix (NECUC) was presented. After total hysterectomy with bilateral salpingo-oophorectomy and adjuvant chemotherapy, a left renal tumor and a pancreatic lesion developed and were both diagnosed on pathological examination as metastases from NEC. In addition, a brainstem metastasis causing neurologic signs developed. The brain lesion was treated by stereotactic radiotherapy (SRT) and the renal and pancreatic lesions by stereotactic body radiotherapy (SBRT). Despite control of the renal and pancreatic lesions, multiple small lung metastases developed later. Recurrence and newly developed brain metastases were treated by repeat stereotactic radiosurgery (SRS)/SRT successfully. Chemotherapy was continued and controlled the lung metastases until three and a half years after the initial operation of the uterus.

## Introduction

Neuroendocrine tumors (NETs) are uncommon and aggressive malignancies derived from neuroendocrine cells. NETs are typically located in the pancreas, gastrointestinal tract (GI tract), and lungs [1]. Neuroendocrine carcinoma (NEC) is a poorly differentiated type of NET. Rarely, NECs may also occur in other organs such as the female genitalia. NEC of the uterine cervix (NECUC) is a rare malignancy constituting fewer than 3% of all cervical tumors [1,2]. NECUC are common in perimenopausal females and present with abnormal vaginal bleeding and mimic endocervical carcinomas, usually with no distinguishing symptoms. However, the biology of NECUC differs from that of squamous cell carcinoma or adenocarcinoma of the cervix in a number of aspects. For example, NECUC is more likely to have invaded the lymphovascular space and to spread to the regional lymph node basin at the time of diagnosis. Local and distant relapses also occur more often in NECUC, and the five-year overall survival is significantly poorer at around 30% compared to >65% for squamous cell carcinoma and adenocarcinoma of the cervix [1]. Thus, the aggressive nature of NECUC resembles that of small cell lung cancer that, at the time of initial diagnosis, is rarely localized and mostly locally advanced or metastatic.

We herein report a case of NECUC. After total hysterectomy and bilateral salpingo-oophorectomy with an initial diagnosis of localized NECUC, the patient received adjuvant chemotherapy. On subsequent follow-up, she developed a left renal tumor, pancreatic lesion, and brain metastasis. All of these lesions were successfully treated by stereotactic irradiation. Lung metastases, which developed later, were also controlled with continuing chemotherapy.

## Case presentation

**Figure 1:**
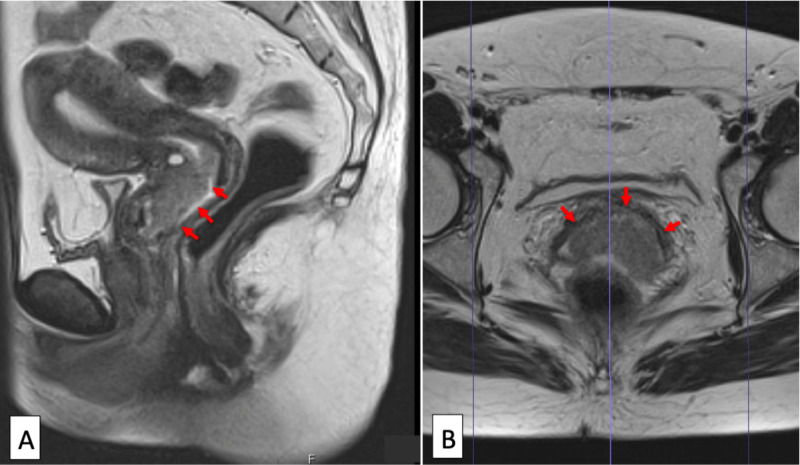
Initial magnetic resonance imaging of uterine cervix tumor Sagittal (A) and axial (B) magnetic resonance images showed an irregular-shaped uterine cervix tumor (arrows).

After a 46-year-old woman complained of metrorrhagia, a uterine cervix tumor was detected (Figure 1).

**Figure 2:**
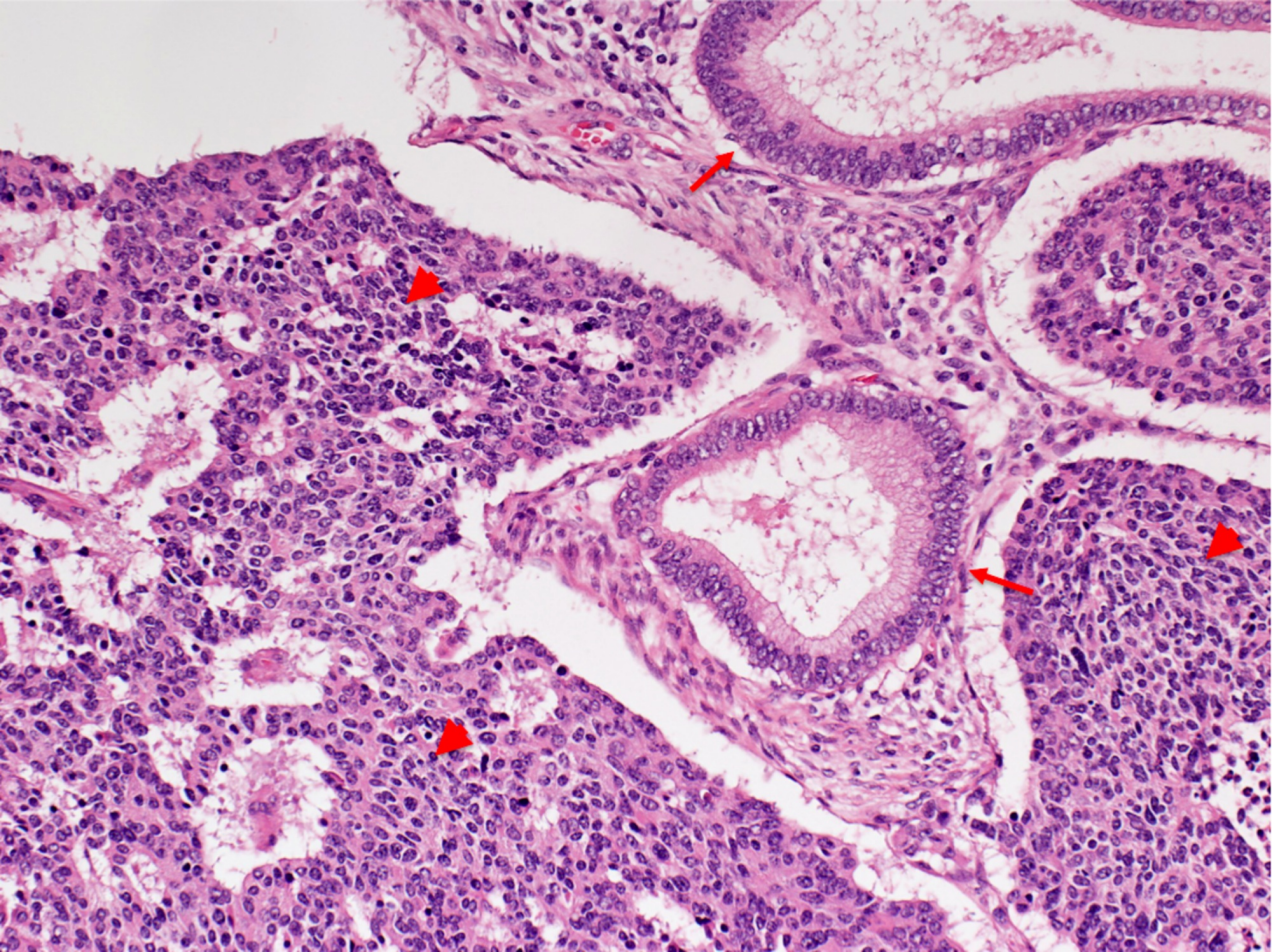
Surgical specimen of uterine cervix tumor (hematoxylin and eosin stain, magnification x200) Coexisting large cell neuroendocrine carcinoma and endocervical carcinoma. Large cell neuroendocrine carcinoma component (arrow heads) is composed of cuboidal cells with high N/C (nucleus/cytoplasm) ratio. Endocervical adenocarcinoma component (arrows) is composed of tall columnar cells.

The cytology was negative for intraepithelial lesion or malignancy. The histopathological results of a colposcopic biopsy revealed endocervical carcinoma suspected with undifferentiated carcinoma component. Total hysterectomy and bilateral salpingo-oophorectomy were performed. Histological diagnosis was large cell NEC (pT1b1N0M0; tumor diameter <4 cm, no lymph node involvement, no distant metastasis) with an endocervical carcinoma component (Figure 2).

**Figure 3:**
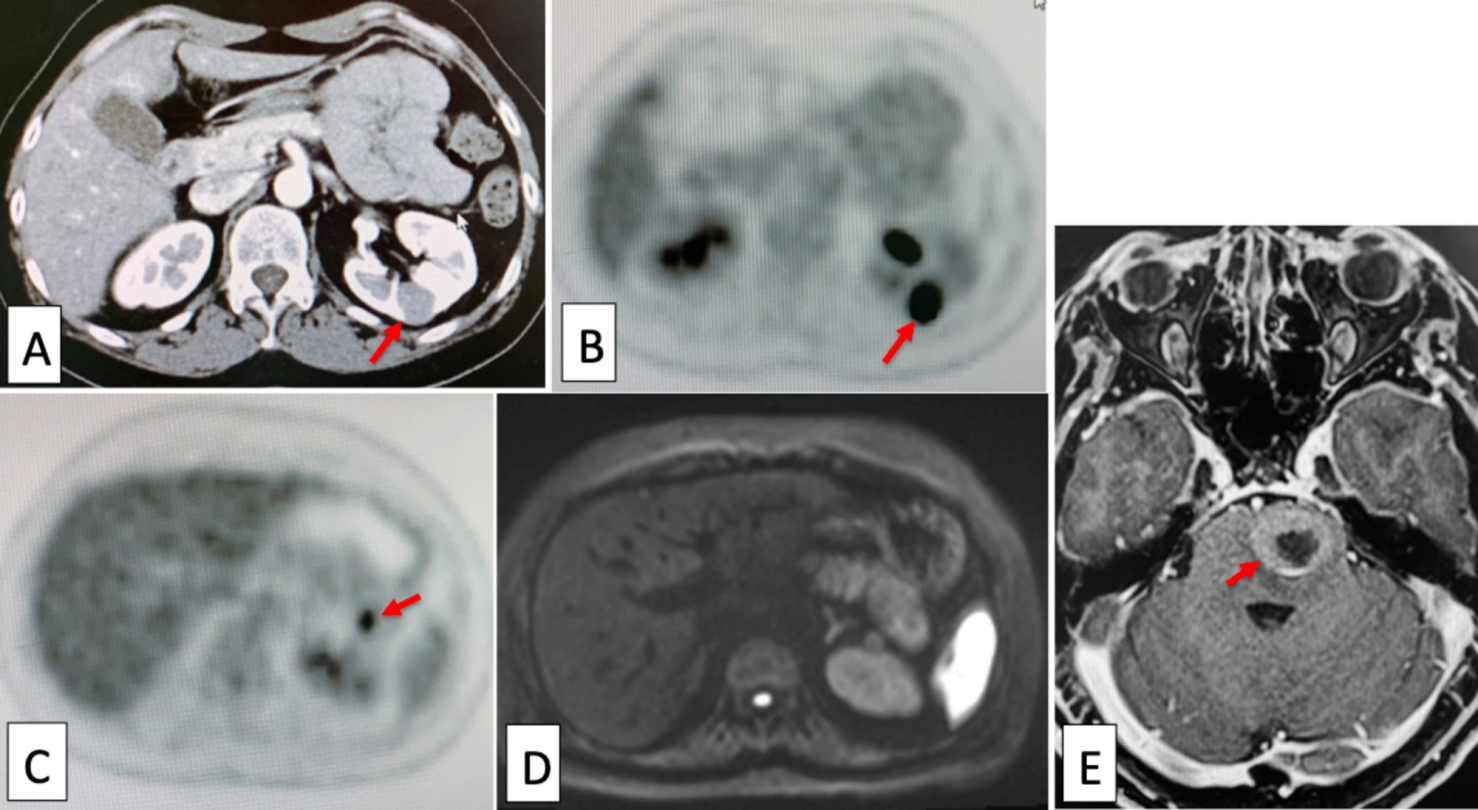
Left kidney, pancreas, and brain metastases Computed tomography (CT) showed a mass in the left kidney (A, arrow), which showed high-uptake of FDG (2-[18F]fluoro-2-deoxy-D-glucose) in positron emission tomography (FDG-PET) (B, arrow). FDG-PET also revealed an FDG high-uptake lesion in the pancreas (C, arrow). The pancreatic lesion was obscure on CT and magnetic resonance imaging (MRI) (D). Brain MRI showed a metastasis with ringed gadolinium contrast enhancement in the pons (E, arrow).

Immunohistochemical study revealed positive results for chromogranin A, synaptophysin, CD (cluster of differentiation) 56, and p16 in the NEC component. Adjuvant chemotherapy with CPT-P (Irinotecan and Cisplatin) was given. One year after the operation, a mass lesion was detected in the left kidney on computed tomography (CT) (Figure 3A). In addition, positron emission tomography with 2-[^18^F]fluoro-2-deoxy-D-glucose (FDG-PET) disclosed FDG-high-uptake tumors in the left kidney (Figure 3B) and pancreas (Figure 3C).

**Figure 4:**
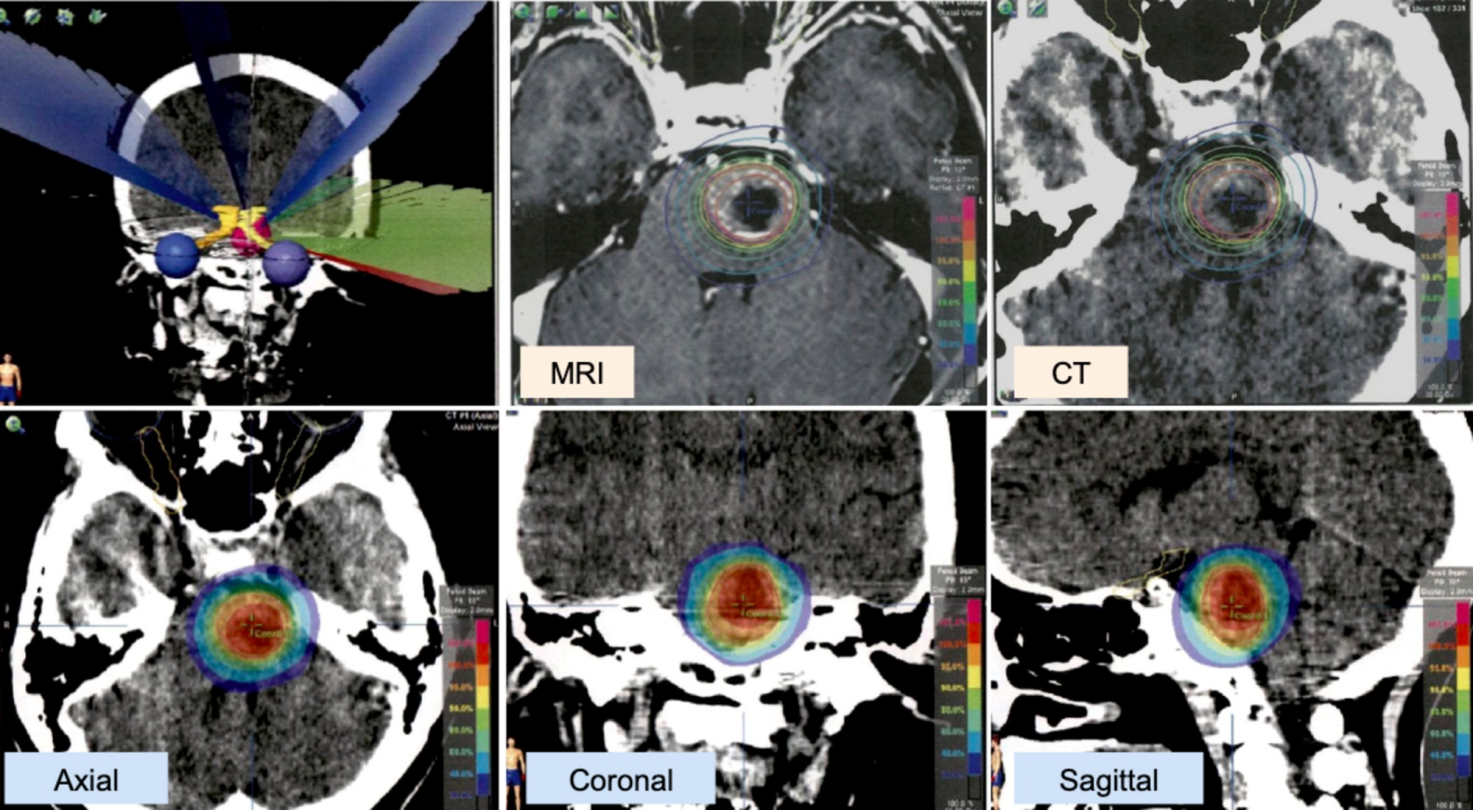
Stereotactic radiotherapy for brain metastasis Stereotactic radiotherapy (SRT) treatment planning on iPlan (BrainLAB, Tokyo) workstation. A dose of 30 Gy in five fractions (D95 [dose to 95% of the planning target volume (PTV)] = 95% dose) was delivered to the brainstem metastasis (PTV = 7.0 ml) by dynamic conformal multi-arc SRT using TrueBeamSTx (Varian, Tokyo).

Both tumors were diagnosed as NEC metastases by biopsy. Tumor cells of both lesions were chromogranin A, CD56, and synaptophysin positive. Around the same period, she developed dysarthria and gait disturbance due to right-sided leg motor weakness, which was caused by a brainstem metastasis (Figure 3E). This was treated by lineac-based stereotactic radiotherapy (SRT, 30 Gy/5 fractions [fx.]) with TrueBeamSTx (Varian, Tokyo) with ExacTrac system (BrainLAB, Tokyo) (Figure 4).

**Figure 5:**
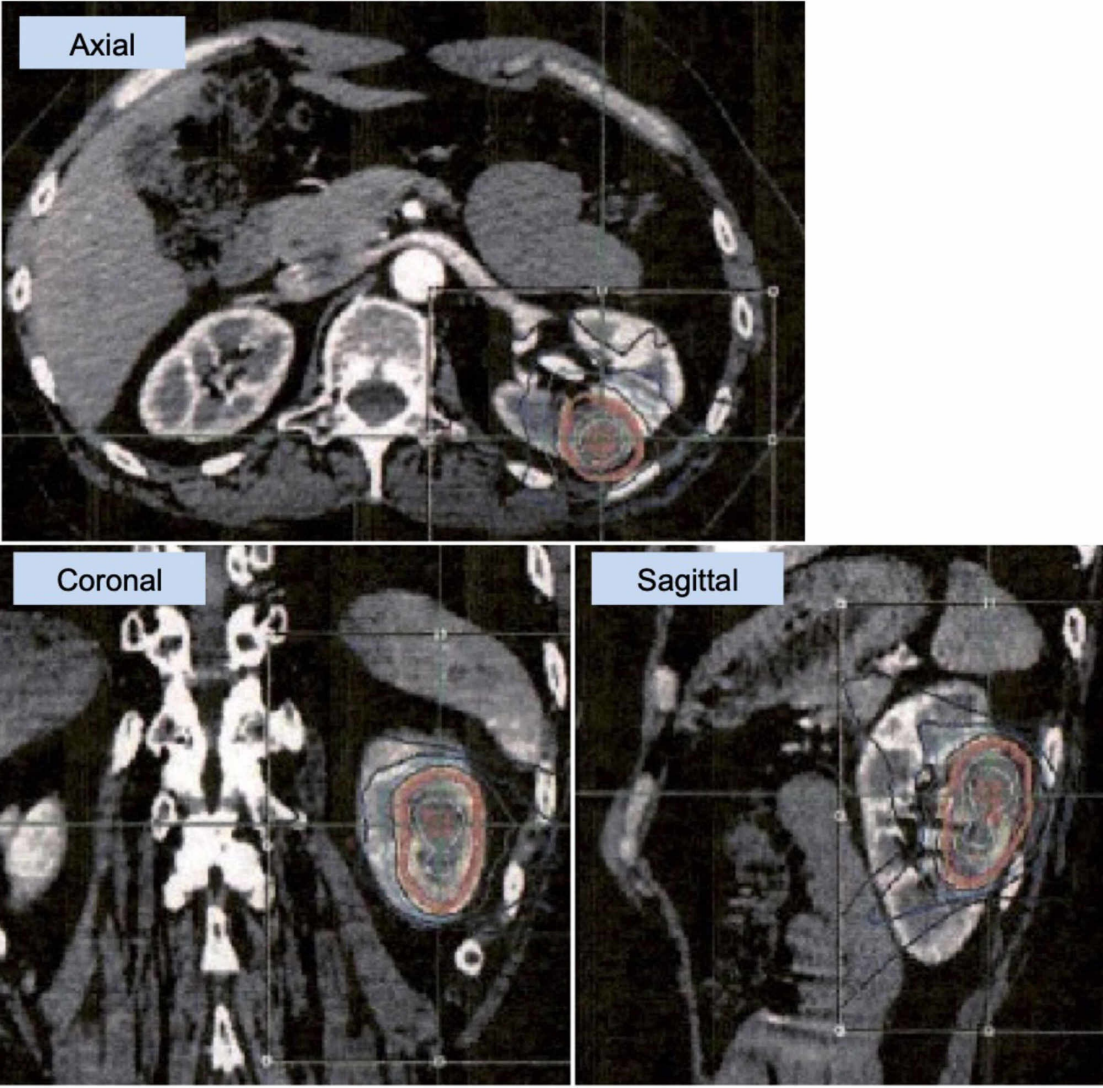
Stereotactic body radiotherapy for renal metastasis Stereotactic body radiotherapy (SBRT) treatment planning on MultiPlan (Accuray, Tokyo) workstation. A dose of 35 Gy in five fractions (D95 = 100% dose) was delivered to the left kidney metastasis (PTV = 19.9 ml) by CyberKnife (Accuray, Tokyo).

**Figure 6:**
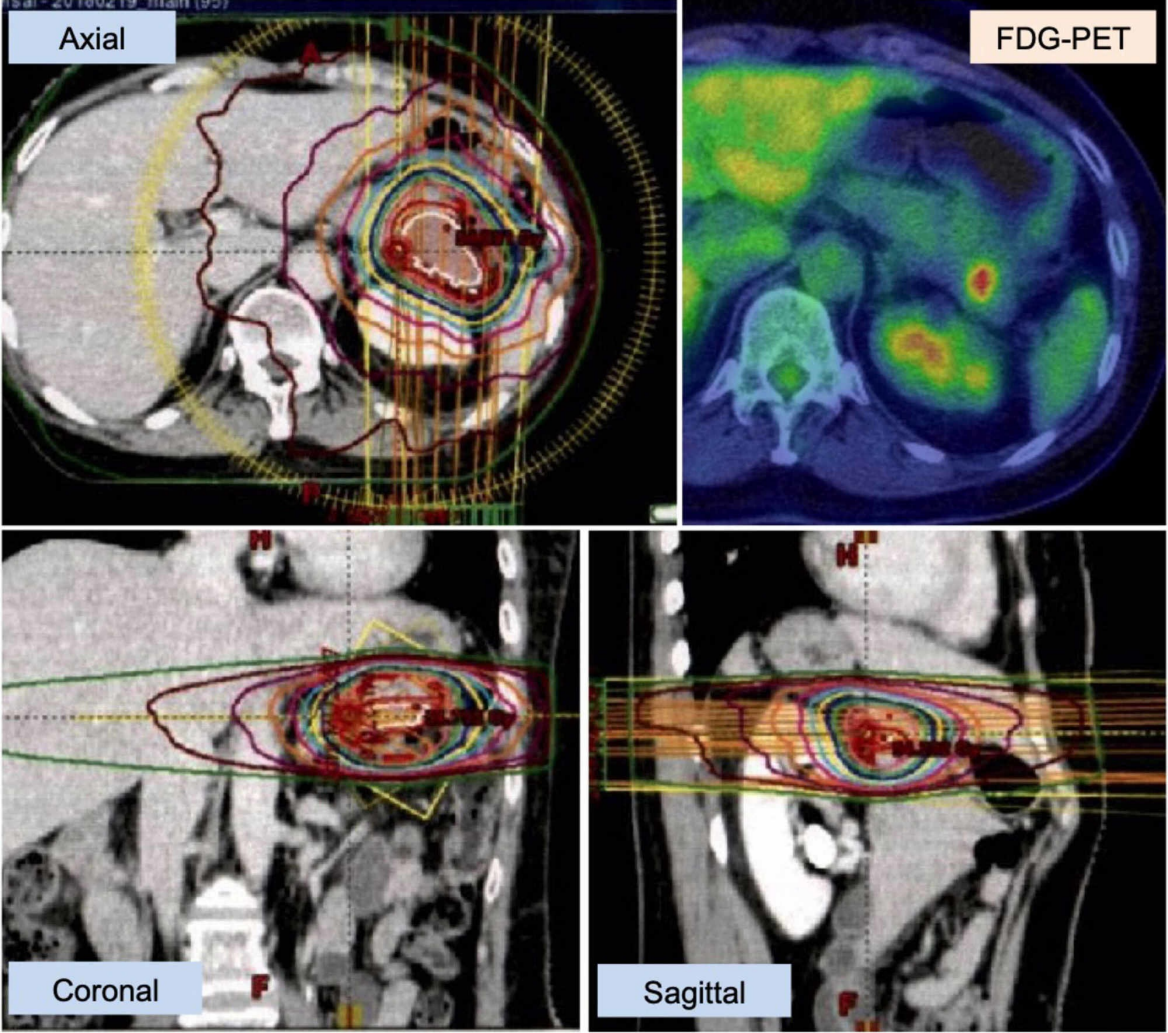
Stereotactic body radiotherapy for pancreatic metastasis Stereotactic body radiotherapy (SBRT) treatment planning on Ecripse (Varian, Tokyo) workstation. A dose of 50 Gy in 20 fractions (D95 = 100% dose) was delivered to the pancreatic metastasis (PTV = 51.5 ml) by VMAT (volumetric modulated arc therapy) using TruebeamSTx (Varian, Tokyo).

Both the left renal tumor and pancreatic lesion were treated with stereotactic body radiation therapy (SBRT). The renal metastasis was treated with CyberKnife (Accuray, Tokyo) SRT (35 Gy/5 fx.) (Figure 5) and the pancreatic lesion with volumetric-modulated arc therapy (VMAT)-SRT (50 Gy/25 fx.) using TrueBeamSTx (Figure 6).

Later, multiple small lung metastases developed and chemotherapy with pacritaxel and cisplatin or bevacizumab was continued. In addition, she developed new brain metastases and brainstem metastasis recurrence, which were treated by stereotactic radiosurgery (SRS) (20-21 Gy) and SRT (24 Gy/3 fx.) with Gamma Knife with eXtend system (Elekta, Tokyo). Totally 10 brain tumors were treated, including three treated twice, and all were controlled. Both the renal and pancreatic metastases disappeared. The lung lesions were also stable at the end of the follow-up. Her condition remained stable with only slight motor weakness in the left leg until three and a half years after the initial operation of the uterus.

## Discussion

NETs are subdivided into well-differentiated NETs, including NET grade 1 (also known as typical carcinoid) and NET grade 2 (also known as atypical carcinoid), and poorly differentiated NEC [3]. NECUC is a rare aggressive histological variant of cervical cancer. Ishikawa et al. summarized the cases of NETs of UC in a multi-center retrospective study [4]. Among total 193 cases, diagnoses were atypical carcinoid tumor in 37, small cell NEC in 126, large cell NEC in 22, and NET, and otherwise classified in eight. There is no standard chemotherapy regimen for NECUC, and the regimens similar to the treatment routinely used for small cell lung cancer are selected [1]. Cisplatin/carboplatin with etoposide (EP) has been the most commonly used treatment regimen. EP combined with other agents such as bleomycin, cyclophosphamide, or doxorubicin has also been reported [1,4]. Other commonly used cytotoxic regimens in the primary therapy setting (neoadjuvant or adjuvant) are cisplatin/carboplatin and paclitaxel and cisplatin combined with irinotecan. Other regimes such as 5-fluorouracil/cisplatin, vincristine/cisplatin/bleomycin, vincristine/adriamycin/cisplatin, and irinotecan/cisplatin/paclitaxel have only rarely been used [1]. In women with recurrent NECUC, EP alone or in combination with other cytotoxic drugs has also been the most commonly used cytotoxic regimen. Targeted therapies and immune-checkpoint inhibitors have also been tried [1]. In the present case, chemotherapy has been continued with similar regimens for adjuvant after the initial surgery and for salvage after development of bilateral lung metastases.

In the present case, we administered stereotactic irradiation to all of the three lesions that developed after the initial surgery of the primary uterine lesion and following adjuvant chemotherapy. Stereotactic irradiation delivers a higher biological effective dose to the tumor with sharp dose escalation in a shorter treatment time course. The efficacy of SRS/SRT for brain metastases has been well established [5,6]. In the present case, all brain lesions have been controlled so far, though three of the 10 lesions required treatment twice. Recently, more and more cases have been treated by SBRT for primary or metastatic body lesions. Some papers have been published on SBRT for primary pancreatic carcinoma [7-9]. Wei et al. summarized the treatment results of SBRT for pancreatic cancer, and listed the reported series of SBRT [9]. The delivered dose varied among reported series, 15-25 Gy in a single session and 24-45 Gy in 3-5 fractions. In this case we used a rather conservative fractionated SBRT using VMAT (50 Gy in 20 fractions), out of concern for possible adverse effects to the duodenum adjacent to the radiation field. Some papers have also been published on SBRT for primary renal cell carcinoma. Correa et al. reviewed the treatment results of SBRT for primary renal cell carcinoma [10]. In addition, recently some studies on SBRT for oligometastases have become available [11,12]. Suh and Cho showed the results in metastases from non-small-cell lung carcinoma (NSCLC) [11]. Sutera et al. showed the results in metastases from various malignancies (NSCLC, SCLC, colorectal adenocarcinoma, head and neck, squamous cell carcinoma, breast carcinoma and prostate adenocarcinoma) [12]. In our patient, all the detected lesions, brain, left kidney, and pancreas metastases, were treated by stereotactic irradiation at that time. Later, tiny bilateral lung metastases developed but have not deteriorated with continuing chemotherapy.

The present case developed only renal and pancreatic lesions after the surgery of the primary uterine lesion followed by adjuvant chemotherapy. Metastatic pancreatic cancers are rare neoplasms accounting for approximately 2-5% of all pancreatic tumors [13]. Adsay et al. investigated the primary origin of secondary tumors of the pancreas [14]. In 81 autopsy cases, the origin of the secondary pancreatic lesion was lung (34 cases), GI tract (20), kidney (four), breast (three), liver (two), ovary (one), and urinary bladder (one). In addition, tumors of hematopoietic origin (six), melanomas (two), sarcomas (two), and mesotheliomas (two) were also seen. In 38 surgical specimens, it was stomach (seven), kidney (six), lung (two), liver (one), prostate (one), ovary (one), uterus (one), Merkel cell carcinoma (one), malignant gastrointestinal stromal tumors (three), sarcoma (one), and lymphomas (11). As for renal metastases, Sanchez-Ortiz et al. reviewed the renal mass histology in 100 consecutive clinical patients [15]. The primary malignant types were GI-tract (15), lung (13), breast (13), prostate (six), pancreas (five), head and neck (five), ovary (two), testis (two), uterus (one), skin (one), biliary (one), bladder (one), melanoma (eight), neuroblastoma (one), lymphoma (22), myelodysplasia (two), and extremity sarcoma (two). Uterus origin was infrequent in both pancreatic metastasis and renal metastasis. Considering NEC, Sekine et al. reported two cases of genital large cell NEC that had renal metastases as well as miliary lung metastasis [16]. Mackay et al. reported two cases of small cell NECUC with involvement of the pancreas [17]. Kopke Tulio et al. reported a case of NECUC whose initial manifestation was pancreatic metastases [18]. Lee et al. also reported a case of small cell NEC with pancreatic metastasis [19]. Meanwhile, Kawasaki et al. reported a case of renal NET with synchronous pancreas metastasis [20].

## Conclusions

A rare case of NECUC developing renal, pancreatic, and brain lesions, after surgery of the primary site and adjuvant chemotherapy, was described. All of these lesions were treated with stereotactic irradiation successfully and chemotherapy for lung metastases was continued to keep the patient’s condition stable. Local treatment of stereotactic irradiation, with systemic chemotherapy, might be beneficial to control the disease.
